# Non-Linear Associations Between Serum Vitamin D and Uric Acid in Korean Adults: 2022–2023 KNHANES Data

**DOI:** 10.3390/nu17152398

**Published:** 2025-07-22

**Authors:** Hyang-Rae Lee, Nam-Seok Joo

**Affiliations:** Department of Family Practice and Community Health, Ajou University School of Medicine, Suwon 16499, Republic of Korea; hyangle1@naver.com

**Keywords:** vitamin D, uric acid, metabolic disorders, aging, nonlinear association

## Abstract

**Objectives:** This study aimed to investigate both the linear and non-linear associations between serum 25-hydroxyvitamin D [25(OH)D] levels and serum uric acid concentrations in Korean adults, with a particular focus on the vitamin D-insufficient range (<30 ng/mL), and to explore the potential metabolic implications of this relationship. **Methods:** Using data from the Korea National Health and Nutrition Examination Survey (KNHANES), we analyzed 10,864 adults aged 19 years and older. Serum vitamin D levels were categorized into quartiles (Q1–Q4), and their relationships with uric acid concentrations were examined using Pearson correlation, analysis of variance (ANOVA), and restricted cubic spline regression. Multivariate models were adjusted for potential confounders including age, sex, body mass index (BMI), kidney function, chronic disease status, and macronutrient intake. **Results:** In unadjusted analysis, a statistically significant but weak negative correlation was observed between serum 25(OH)D and uric acid levels (Pearson’s r = −0.092, *p* < 0.001). However, in multivariate regression adjusting for confounders, a weak positive association emerged. Restricted cubic spline analysis revealed significant positive associations in the lower quartiles (Q1–Q3), with the strongest association in Q3 (β = 0.769, 95% CI: 0.34–1.19, *p* < 0.001). No significant association was observed in the highest quartile (Q4). **Conclusions:** Serum vitamin D and uric acid concentrations show a non-linear relationship, with a significant positive association within the vitamin D-insufficient range (<30 ng/mL). These findings provide new insights into the potential metabolic role of vitamin D and highlight the need for longitudinal and interventional studies to clarify causality and clinical significance.

## 1. Introduction

Serum uric acid plays a critical role in the regulation of inflammation and oxidative stress and is implicated in the pathophysiology of various chronic metabolic diseases, including cardiovascular disease, metabolic syndrome, obesity, and type 2 diabetes [1,2,3]. Meanwhile, vitamin D, which is well known for its role in metabolic regulation, may influence uric acid metabolism through its anti-inflammatory effects, improvement of insulin sensitivity, attenuation of oxidative damage, and modulation of immune responses [4,5,6].

Recent studies have suggested that vitamin D and uric acid may interact through shared physiological mechanisms such as immune modulation and renal function [7,8]. In particular, vitamin D deficiency may promote the production of pro-inflammatory cytokines such as interleukin-6 (IL-6) and tumor necrosis factor-alpha (TNF-α), leading to impaired kidney function and subsequent uric acid accumulation. Conversely, hyperuricemia may activate the NLRP3 inflammasome, exacerbate inflammation, and disrupt vitamin D metabolism [9,10,11].

However, existing studies examining the association between vitamin D and uric acid have reported inconsistent findings, including positive, negative, and non-linear associations, likely due to differences in study design, population characteristics, and environmental factors [12,13,14]. Although sufficient vitamin D levels have generally been associated with lower serum uric acid concentrations, the nature of this relationship under vitamin D-insufficient conditions remains unclear, with inconsistent findings across studies [13,15].

Some recent meta-analyses have suggested the possibility of a bidirectional association between vitamin D and uric acid, indicating that multiple physiological pathways may be involved [16,17]. Additionally, prior studies have proposed that demographic factors such as age and sex may contribute to the observed variability in this association [12,18]. Given the high prevalence of vitamin D insufficiency in Korea—largely attributed to limited sunlight exposure and predominantly indoor lifestyles—this population provides an ideal epidemiological setting to further investigate the association under insufficient vitamin D conditions, which remain insufficiently explored in prior research [19,20,21].

Therefore, using nationally representative data from the Korea National Health and Nutrition Examination Survey (KNHANES), this study aimed to examine the nature of the association between serum 25-hydroxyvitamin D [25(OH)D] and serum uric acid levels. We also conducted subgroup analyses by age and sex to determine whether these associations vary across demographic groups. By addressing inconsistencies in the existing literature and exploring whether the relationship follows a linear or non-linear pattern, our study offers a more refined perspective on the link between vitamin D status and uric acid metabolism.

## 2. Materials and Methods

### 2.1. Study Population

This study utilized data from the Korea National Health and Nutrition Examination Survey (KNHANES) to examine the association between serum vitamin D levels and serum uric acid concentrations. KNHANES, conducted annually by the Korea Disease Control and Prevention Agency (KDCA), is a nationally representative cross-sectional survey designed to assess the health and nutritional status of the Korean population. Each survey cycle employs a complex, stratified, multistage probability sampling method to select an entirely new, independent sample, thereby representing a cross-section of the Korean population at the time of the survey. Notably, no longitudinal follow-up is conducted across cycles.

**Figure 1:**
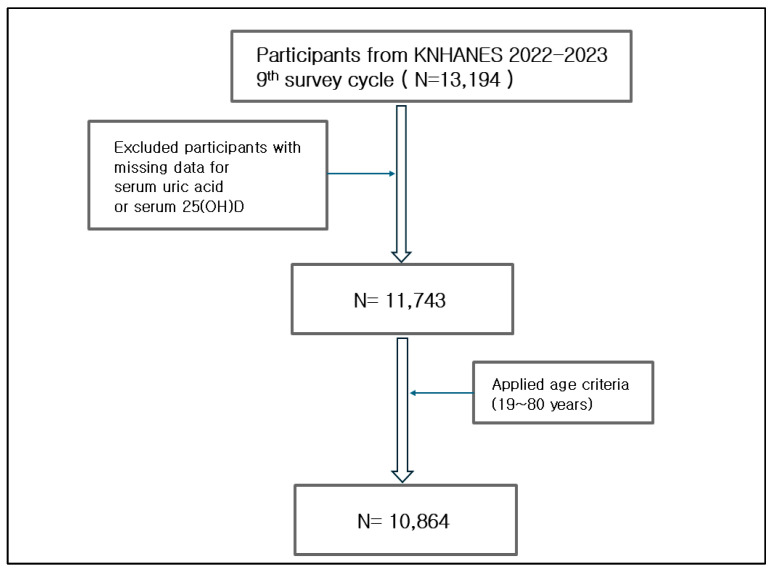
Flowchart of participant selection. This figure outlines the selection process for study participants from the 9th KNHANES (2022–2023), starting with 13,194 individuals. After excluding those with missing data (serum uric acid or 25(OH)D levels), 11,743 participants remained. Applying the age criterion (19–80 years) further reduced the sample to 10,864 participants, who were included in the final.

Since the 9th cycle (2022–2023), KNHANES has standardized the measurement of serum 25-hydroxyvitamin D [25(OH)D] using liquid chromatography-tandem mass spectrometry (LC-MS/MS), improving measurement accuracy and international comparability. Because the assay method was revised beginning with the 9th cycle, the results are not directly comparable to those from previous cycles. Therefore, this study focused on data from the first two years of the 9th cycle (2022–2023), in which both serum vitamin D and uric acid levels were measured. The analysis was restricted to adults aged 19 years or older. Participants with missing key data such as age, sex, serum uric acid, or vitamin D levels were excluded to maintain the integrity of the dataset while preserving sample size. After applying these exclusion criteria, a total of 10,864 participants were included in the final analysis (Figure 1).

As part of the 2022–2023 KNHANES protocol, all participants provided written informed consent prior to participation. This study was conducted in accordance with the principles of the Declaration of Helsinki. Since the KNHANES datasets are publicly available, anonymized, and de-identified, this secondary analysis was exempt from institutional review board approval (IRB Approval Number: AJOUIRB-EX-2024-629).

### 2.2. Clinical and Laboratory Variables

Serum total 25-hydroxyvitamin D [25(OH)D] concentrations, calculated as the sum of 25(OH)D_2_ and 25(OH)D_3_, and serum uric acid levels were obtained from the KNHANES database. Demographic variables included age (years) and sex (male or female). Anthropometric measures comprised body mass index (BMI, kg/m^2^), waist circumference (WC, cm), and blood pressure levels [systolic (SBP, mmHg) and diastolic (DBP, mmHg)]. Medical variables encompassed the prevalence of hypertension, diabetes, and dyslipidemia, determined via questionnaire-based responses. Laboratory markers measured in a fasting state included fasting glucose, glycated hemoglobin (HbA1c), lipid profiles (total cholesterol, triglycerides, low-density lipoprotein cholesterol [LDL-C], and high-density lipoprotein cholesterol [HDL-C]), creatinine (Cr), and high-sensitivity C-reactive protein (hs-CRP, mg/L). Lifestyle variables included alcohol consumption frequency and daily nutrient intake—specifically protein, carbohydrates, fat, and micronutrients such as vitamins C, E, and A—assessed using validated 24 h dietary recall. Physical activity was also included and defined according to the World Health Organization (WHO) guidelines for aerobic exercise, which recommend engaging in at least 150 min of moderate-intensity or 75 min of vigorous-intensity aerobic activity per week.

### 2.3. Statistical Analysis

To examine the distribution of key variables, particularly serum total 25-hydroxyvitamin D [25(OH)D] and uric acid, we conducted exploratory analyses using histograms and density plots (Figures S1 and S2) and calculated skewness before and after log-transformation (Table S1). Log-transformation reduced the skewness of 25(OH)D (from 0.933 to −0.335), but we retained the original non-transformed values to preserve clinical interpretability based on commonly used thresholds (e.g., 20 or 30 ng/mL). Sensitivity analyses using log-transformed values confirmed that the direction and significance of the findings were preserved.

To assess the influence of outliers, we compared two definitions: (A) excluding values above 100 ng/mL based on clinical toxicity [22,23], and (B) excluding values beyond ±3 standard deviations. As approach B excluded participants within the clinically relevant 50–70 ng/mL range, we selected the 100 ng/mL cutoff. The distributions are shown in Figure S2.

**Figure 2:**
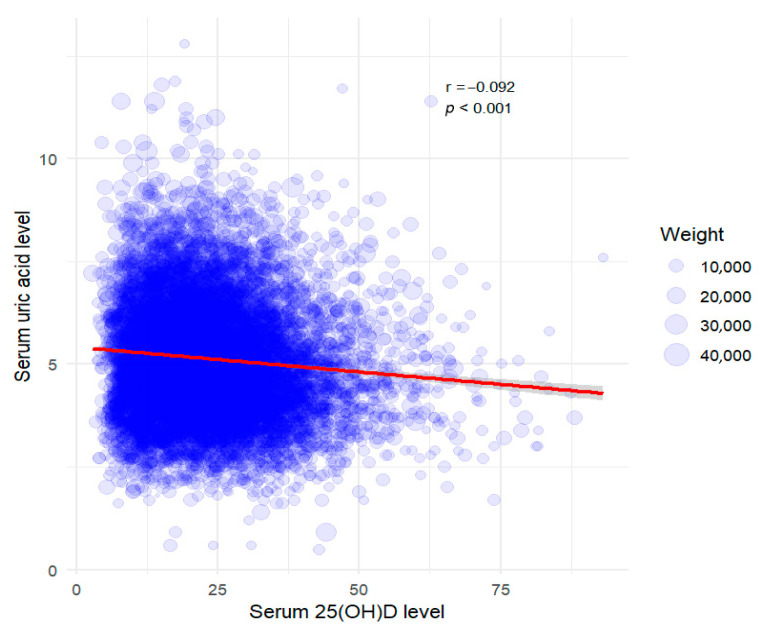
Weighted scatter plot with regression line for serum 25(OH)D vs. serum uric acid. The red line represents the linear regression model, accounting for sampling bias with applied weights. Pearson’s correlation coefficient (PCC) was −0.092 (*p*-value < 0.001). Data points are scaled according to sampling weights.

After excluding outliers, a total of 10,859 participants remained in the final analytic sample. We used multiple statistical approaches to analyze the association between serum total 25(OH)D and uric acid levels. A scatterplot was used to visualize the association, and Pearson’s correlation coefficient (PCC) was calculated to assess linearity (Figure 2). Serum 25(OH)D levels were categorized into quartiles (Q1–Q4), and one-way analysis of variance (ANOVA) was conducted to compare mean uric acid levels across quartile groups. Post hoc pairwise comparisons were performed using the Bonferroni adjustment, and a *p*-value < 0.05 was considered statistically significant.

Multivariate linear regression analysis was conducted to adjust for potential confounding variables, including age, sex, BMI, 24 h dietary intake (carbohydrates, fat, protein, and vitamins C, E, and A), physical activity (defined according to WHO guidelines), and other clinical factors.

To evaluate potential non-linear associations between serum 25(OH)D and uric acid levels, we applied restricted cubic spline regression using covariates included in Model 3—age, sex, BMI, macronutrient intake, and clinical factors. Notably, dietary antioxidant intake and physical activity, which were included in Model 4 for sensitivity analyses, were excluded from the main spline models to maintain consistency with the primary analysis and avoid overadjustment. Although Model 4 showed the lowest AIC and BIC values (Table S2), these variables were considered potential mediators in the causal pathway between vitamin D status and uric acid levels. Therefore, Model 3 was selected for the main analyses to better capture the direct association, while Model 4 was used in sensitivity analyses to confirm the robustness of the findings.

In parallel, generalized additive models (GAMs) were employed to flexibly capture potential non-linear trends using Model 3 covariates. To determine the best-fitting model, we compared spline models with different knot numbers using Akaike Information Criterion (AIC) and Bayesian Information Criterion (BIC) (Table S3).

Subgroup analyses were further conducted by stratifying participants based on combinations of sex, age group, and BMI category to explore possible effect modification.

## 3. Results

### 3.1. Baseline Characteristics

Baseline characteristics by serum total 25-hydroxyvitamin D [25(OH)D] quartiles are presented in Table 1, summarizing demographic, clinical, and biochemical variables. As 25(OH)D levels increased, participants were generally older, more likely to be female, and had higher systolic blood pressure. Conversely, higher vitamin D levels were associated with lower body mass index (BMI), waist circumference (WC), triglycerides, low-density lipoprotein cholesterol (LDL-C), serum uric acid levels, and the proportion of individuals reporting monthly alcohol consumption (≥1 drink/month). No significant differences were observed in high-sensitivity C-reactive protein (hs-CRP) levels across quartiles. One-way ANOVA revealed significant differences in mean serum uric acid levels among vitamin D quartiles (*p* < 0.001), and Bonferroni-adjusted post hoc tests showed that Q4 had significantly lower levels than Q1–Q3.

### 3.2. Linear Correlation Analysis

A scatterplot revealed a weak negative correlation between serum 25(OH)D level and uric acid concentration (Pearson’s correlation coefficient [PCC]: −0.092, *p* < 0.001; Figure 2). This finding suggests that higher serum 25(OH)D levels might be associated with slightly lower uric acid concentrations. However, the correlation strength was minimal and likely influenced by additional factors.

In contrast, multivariate analysis using weighted data showed no significant association after adjusting for age and sex (Model 1; Table 2). When BMI was added to the adjustment (Model 2), a significant but very weak positive correlation emerged, differing from the initial negative trend observed in the scatterplot. Further adjustments for alcohol use, creatinine, chronic disease status (hypertension and diabetes), and lipid profiles (HDL-C, triglycerides, LDL-C) in Model 3 yielded a statistically significant result, but the association remained weak and positive. These findings underscore the complexity of the relationship between serum 25(OH)D and uric acid, and highlight the necessity of thorough adjustment for potential confounders.

To comprehensively adjust for additional confounding factors, we developed a fully adjusted model (Model 4; Table 2) that incorporated the intake of antioxidant vitamins (vitamins C, E, and A [RAE]) based on 24 h dietary recall data, along with aerobic physical activity based on WHO guidelines. Importantly, the association between serum 25(OH)D and uric acid remained statistically significant after these additional adjustments.

Finally, stratified subgroup analyses combining sex with age (<60 vs. ≥60 years; Table S4, Figure S3) and BMI categories (<25 vs. ≥25 kg/m^2^; Table S5, Figure S4) revealed further effect modification across demographic strata. Notably, a statistically significant association was observed in women aged ≥60 years.

### 3.3. Non-Linear Analysis

Given the discrepancy between the initial scatterplot and multivariate models, we applied restricted cubic spline analysis to further examine the potential non-linear relationship between serum 25(OH)D and uric acid levels. As the study focused on vitamin D insufficiency, we specifically investigated whether this association was more evident at lower vitamin D concentrations.

**Figure 3:**
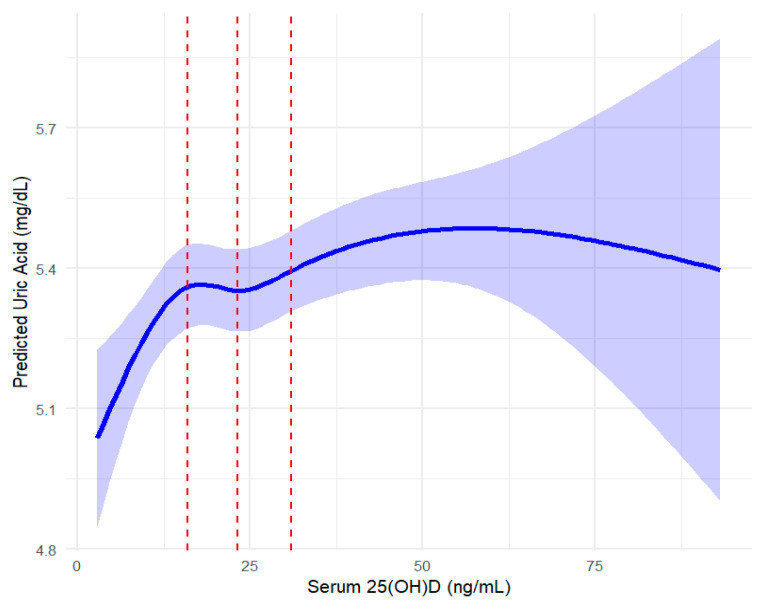
Spline regression analysis of serum 25(OH)D and serum uric acid with confidence intervals. The curve represents the restricted cubic spline regression model, with shaded areas indicating 95% confidence intervals. Red vertical lines divide quartiles (Q1: <15.96 ng/mL, Q2: 15.96–23.2 ng/mL, Q3: 23.2–30.92 ng/mL, Q4: >30.92 ng/mL) of serum 25(OH)D concentrations. This figure provides a visual representation of the results presented in Table 3.

The spline regression was conducted with sampling weights and adjusted for covariates in Model 3, including age, sex, BMI, alcohol consumption, serum creatinine, chronic disease status (hypertension, diabetes, dyslipidemia), dietary intake (carbohydrates, fats, proteins), and lipid profiles (HDL-c, TG, LDL-c). Significant positive associations were observed in Q1 (<15.96 ng/mL), Q2 (15.96–23.2 ng/mL), and Q3 (23.2–30.92 ng/mL), with the strongest effect in Q3 (β = 0.769, 95% CI: 0.34–1.19, *p* < 0.001; Table 3, Figure 3). However, no significant association was found in Q4 (>30.92 ng/mL; β = 0.184, 95% CI: −0.31–0.68, *p* = 0.465), suggesting a plateau at higher vitamin D levels. To determine whether this pattern was driven by the biologically active component of vitamin D, a parallel analysis was performed using serum 25(OH)D_3_ levels. The results closely mirrored those for total 25(OH)D: significant positive associations in Q1 (<15.61 ng/mL), Q2 (15.61–22.88 ng/mL), and Q3 (22.88–30.61 ng/mL), with the largest effect in Q3 (β = 0.708, 95% CI: 0.29–1.13, *p* < 0.001; Table 4). In Q4 (>30.61 ng/mL), the association remained positive but was not statistically significant (β = 0.188, 95% CI: −0.31 to 0.69, *p* = 0.456). These consistent findings reinforce the hypothesis that 25(OH)D_3_ is primarily responsible for the observed relationship. To ensure robustness, sensitivity analyses using a fully adjusted model (Model 4), which additionally accounted for dietary micronutrient intake and physical activity, yielded consistent results (Table 5).

**Figure 4:**
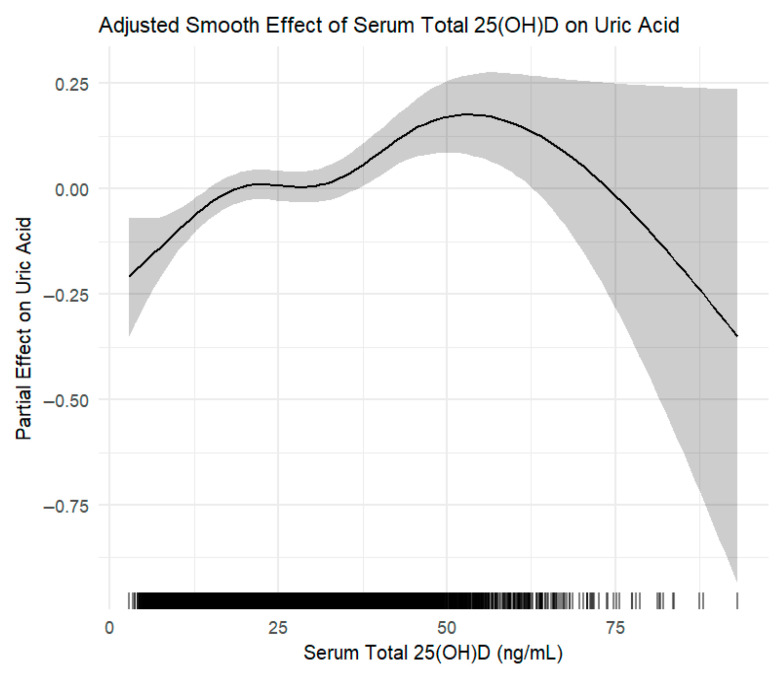
Generalized additive model (GAM) depicting the adjusted smooth effect of serum total 25-hydroxyvitamin D [25(OH)D] on serum uric acid levels. The curve shows the partial effect of 25(OH)D after adjusting for key confounders including age, sex, BMI, alcohol use, creatinine, chronic diseases, dietary intake, and lipid profiles. Shaded area represents the 95% confidence interval.

In addition to restricted cubic spline regression, we applied generalized additive models (GAMs) to flexibly capture the potential non-linear association between serum 25(OH)D levels and uric acid concentrations. The GAM analysis corroborated the spline findings, demonstrating a similar non-linear pattern with a positive association predominantly within the vitamin D insufficiency range and a plateau at higher concentrations (Figure 4).

Stratified spline regression analyses by sex and age (Table S6) revealed that the positive association between serum 25(OH)D concentrations and uric acid levels was most pronounced in women aged 60 years and older (Table 6). This association was consistently significant across the lower-to-mid quartiles of serum 25(OH)D (Q1 to Q3), but not in the highest quartile. A comparable trend was also observed when using serum 25(OH)D_3_ concentrations in this subgroup (Table S7). Among men aged 60 years and older, a similar but less consistent pattern was noted. Further stratification by sex and BMI (Table S8) revealed that statistically significant associations in the lower vitamin D quartiles were more consistently observed in women with a BMI below 25 kg/m^2^. In contrast, women with a BMI ≥25 kg/m^2^ and men generally exhibited weaker or non-significant associations.

## 4. Discussion

This study was conducted under the assumption that the relationship between serum vitamin D levels and uric acid does not follow a simple linear pattern. Accordingly, we focused on the association specifically within the vitamin D-insufficient range, commonly defined as serum 25(OH)D <30 ng/mL. In the overall scatterplot, a weak negative correlation was observed between vitamin D and uric acid levels, supporting the hypothesis that vitamin D may exert anti-inflammatory and metabolic regulatory effects. However, this apparent trend did not persist in fully adjusted models. Multivariate regression analysis adjusting for age, sex, BMI, alcohol use, creatinine, chronic diseases, nutrient intake, and lipid profiles (Model 3) revealed a significant but weak positive association between 25(OH)D and uric acid levels (β = 0.0042, *p* < 0.001), suggesting that this relationship may be influenced by complex metabolic mechanisms. Interestingly, restricted cubic spline regression revealed a significant positive association between serum 25(OH)D and uric acid levels when concentrations were below 30 ng/mL, a threshold widely used to define vitamin D insufficiency. Similar trends were observed for 25(OH)D_3_, the major circulating form of vitamin D. This pattern suggests that the association between vitamin D and uric acid metabolism can vary depending on the concentration of vitamin D, with distinct metabolic impacts in insufficiency (<30 ng/mL) and sufficiency (≥30 ng/mL) ranges. Additionally, generalized additive models (GAMs) were applied to flexibly model the potential non-linear relationship between serum 25(OH)D levels and uric acid concentrations (Figure 4). The GAM analysis corroborated the spline findings, demonstrating a similar non-linear pattern characterized by a positive association primarily within the vitamin D insufficiency range and a plateau at higher vitamin D concentrations.

Our results are in accordance with earlier observations that vitamin D’s metabolic effects may vary depending on serum concentration levels. Previous studies have explored the association between serum vitamin D level and uric acid concentration across diverse populations, emphasizing the complexity and variability of this relationship. For instance, the U.S. National Health and Nutrition Examination Survey (NHANES) to analyze serum 25(OH)D has found that individuals in the lowest quartile of vitamin D levels have a 46% higher risk of hyperuricemia compared to those in the highest quartile [15]. Meanwhile, another cross-sectional study of the general Chinese population has observed a non-linear, inverted U-shaped association between serum 25(OH)D and uric acid concentrations [24]. Additionally, a recent meta-analysis synthesizing existing evidence has suggested that the relationship between vitamin D and uric acid may be complex and potentially bidirectional, rather than linear or unidirectional in nature [16].

Vitamin D is thought to influence uric acid metabolism through several anti-inflammatory and renal mechanisms. It can suppress pro-inflammatory cytokines such as interleukin-6 (IL-6) and C-reactive protein (CRP), thereby stabilizing purine metabolism and reducing uric acid production [25,26]. In addition, vitamin D may indirectly regulate xanthine oxidase activity and modulate renal uric acid handling by downregulating urate transporter 1 (URAT1), while enhancing excretory pathways involving ABCG2 and NPT1/4 transporters [27,28,29]. Paradoxically, we found a positive association between serum vitamin D and uric acid levels among individuals with vitamin D insufficiency (<30 ng/mL). This may reflect a compensatory increase in uric acid—as an endogenous antioxidant—in response to heightened oxidative stress under vitamin D-deficient conditions [30,31]. Furthermore, vitamin D deficiency is associated with elevated parathyroid hormone (PTH) levels, which may increase renal uric acid reabsorption and impair excretion [32,33,34,35]. As vitamin D levels rise within the insufficient range, this may trigger metabolic compensation without fully restoring PTH or renal transporter balance, leading to continued uric acid accumulation [36,37]. These findings suggest that the effect of vitamin D on uric acid metabolism may differ between insufficient and sufficient states.

Notably, this non-linear pattern was most evident in older women (≥60 years), a subgroup in which a particularly strong and consistent association between serum 25(OH)D and uric acid levels was observed from Q1 to Q3 (Table 6). Several aging-related physiological mechanisms may help explain this observation. First, the postmenopausal decline in estrogen levels is known to exacerbate systemic inflammation and oxidative stress, potentially sensitizing uric acid metabolism to shifts in vitamin D status [38]. Estrogen deficiency has also been linked to increased xanthine oxidase activity and altered renal urate excretion, mechanisms that could amplify uric acid accumulation in vitamin D-insufficient states [39,40]. Second, renal function tends to decline with age, and this deterioration may be accompanied by altered expression or function of urate transporters (e.g., URAT1, ABCG2), rendering older adults more susceptible to disruptions in uric acid clearance [29]. Third, age-related alterations in VDR density may modulate tissue responsiveness to changes in circulating vitamin D levels [41]. These factors may interact to amplify the association between vitamin D status and uric acid in this subgroup, highlighting the need to account for age- and sex-specific differences in future research and clinical assessment.

Given the inconsistent findings in previous studies on the association between serum vitamin D and uric acid levels, this study aimed to clarify the relationship by analyzing data from KNHANES, a large-scale, nationally representative survey of Korean adults. The use of this robust dataset enhances the generalizability and external validity of the findings, while the separate evaluation of 25(OH)D_3_, the biologically active form of vitamin D, adds physiological relevance. Furthermore, the identification of a stronger association in older women suggests the potential for age- and sex-specific differences, highlighting the need for individualized clinical approaches. Despite its strengths, this study has several limitations. First, the cross-sectional design precludes causal inferences, making it unclear whether vitamin D could directly influence uric acid levels or if the relationship is bidirectional. Second, residual confounding cannot be ruled out despite adjustments for multiple factors, as unmeasured variables such as physical activity, dietary purine intake, or genetic polymorphisms might have influenced our results. Third, vitamin D levels were measured only once, and the lack of data on sample collection timing means seasonal fluctuations could not be considered [42]. Fourth, while non-linear patterns were identified, the underlying biological mechanisms remain speculative and require further exploration. Finally, as information on the use of vitamin D supplements and uric acid–lowering medications was not available in the KNHANES dataset, the possibility of residual confounding related to these unmeasured factors cannot be fully excluded. However, since serum 25(OH)D and uric acid levels were directly measured as part of the KNHANES protocol and analyzed as continuous variables in this population-based cross-sectional study, our primary aim was to evaluate their association irrespective of the source of exposure or intervention. This inherent limitation is common in large-scale epidemiological analyses and does not detract from the value of the observed population-level patterns. Therefore, future research should focus on elucidating the molecular mechanisms underlying the relationship between vitamin D and uric acid. This includes investigating the roles of vitamin D receptors (VDR), urate transporters, inflammatory cytokines, and oxidative stress pathways. Additionally, longitudinal studies with repeated measurements of vitamin D and uric acid levels are essential for understanding their interactions and temporal dynamics. Randomized controlled trials (RCTs) are indispensable for evaluating effects of vitamin D supplementation on uric acid levels and metabolic outcomes. These studies will help us validate current findings and provide clearer insights into the role of vitamin D as a biomarker.

## 5. Conclusions

This study revealed a non-linear association between serum vitamin D levels and uric acid concentrations, with a significant positive relationship observed within the vitamin D-insufficient range (<30 ng/mL). These findings suggest that vitamin D may influence uric acid metabolism in a concentration-dependent manner. Further longitudinal and interventional studies are warranted to clarify the underlying mechanisms and evaluate the clinical significance, particularly in populations at risk of vitamin D insufficiency.

## Figures and Tables

**Table 1:** Baseline characteristics of participants by the quartile of serum † 25(OH)D.

Variable	Total (N = 10,864)	Quartiles of Serum † 25(OH)D Levels (ng/mL)				*p* Value
		Q1 (N = 2718) (2.82–15.96)	Q2 (N = 2715) (15.96–23.21)	Q3 (N = 2716) (23.21–30.94)	Q4 (N = 2715) (30.94–128.46)	
Age (years)	53.56 ± 16.88	46.44 ± 0.34 ^a^	51.61 ± 0.32 ^b^	55.75 ± 0.3 ^c^	60.47 ± 0.27 ^d^	<0.001
Male (*n*, %) *	4736 (43.59%)	1255 (46.17%)	1332 (49.06%)	1265 (46.58%)	884 (32.56%)	<0.001
BMI (kg/m^2^)	24.07 ± 3.72	24.32 ± 0.08 ^a^	24.35 ± 0.07	24.1 ± 0.07 ^b^	23.5 ± 0.06 ^b^	<0.001
WC (cm)	84.04 ± 10.8	84.13 ± 0.23 ^a^	84.81 ± 0.21	84.39 ± 0.19	82.85 ± 0.19 ^b^	<0.001
SBP (mmHg)	119.6 ± 16.04	118.4 ± 0.31 ^a^	119.39 ± 0.31 ^a^	120.09 ± 0.3	120.52 ± 0.31 ^b^	<0.001
DBP (mmHg)	73.95 ± 9.59	73.8 ± 0.19	74.26 ± 0.19	74.13 ± 0.18	73.6 ± 0.18	0.048
≥1 drink/month (*n*, %) *	5466 (51.01%)	1511 (56.38%)	1465 (54.5%)	1398 (52.3%)	1092 (40.82%)	<0.001
Glucose (mg/dL)	101.21 ± 23.09	101.04 ± 0.49	101.53 ± 0.47	101.55 ± 0.43	100.73 ± 0.38	0.492
HbA1c (%)	5.63 ± 0.79	5.56 ± 0.02 ^a^	5.64 ± 0.02 ^a^	5.63 ± 0.01	5.69 ± 0.01 ^b^	<0.001
T. chol (mg/dL)	186.38 ± 40.46	186.31 ± 0.76	187.31 ± 0.76	186.71 ± 0.78	185.18 ± 0.81	0.262
HDL-c (mg/dL)	57.21 ± 15.49	56.11 ± 0.3 ^a^	56 ± 0.29	56.84 ± 0.29 ^a^	59.9 ± 0.31 ^b^	<0.001
Triglycerides (mg/dL)	126.76 ± 96.14	131.99 ± 1.99 ^a^	130.52 ± 1.82 ^a^	126.85 ± 2.05	117.68 ± 1.46 ^b^	<0.001
LDL-c (mg/dL)	113 ± 36.79	113.36 ± 0.69 ^a^	114.76 ± 0.7 ^a^	113.63 ± 0.71	110.24 ± 0.72 ^b^	<0.001
Creatinine (mg/dL)	0.8 ± 0.24	0.79 ± 0 ^a^	0.81 ± 0.01 ^b^	0.8 ± 0	0.79 ± 0	0.002
Uric acid (mg/dL)	4.98 ± 1.4	5.04 ± 0.03 ^a^	5.13 ± 0.03 ^a^	4.98 ± 0.03	4.79 ± 0.03 ^b^	<0.001
Hs-CRP (mg/L)	1.5 ± 4.61	1.47 ± 0.08	1.46 ± 0.08	1.49 ± 0.08	1.55 ± 0.11	0.916
Carbohydrate Intake (g/day)	255.96 ± 108	254.86 ± 2.16 ^a^	260.75 ± 2.07 ^b^	259.41 ± 2.04	248.92 ± 2.01 ^a^	<0.001
Fat intake (g/day)	46.8 ± 33.31	48.51 ± 0.67 ^a^	48.59 ± 0.68 ^a^	46.81 ± 0.63 ^a^	43.33 ± 0.58 ^b^	<0.001
Protein intake (g/day)	67.39 ± 34.55	67.03 ± 0.67 ^a^	69.34 ± 0.71 ^b^	68.6 ± 0.65	64.64 ± 0.62 ^a^	<0.001
† 25(OH)D	24.46 ± 11.35	11.8 ± 0.05	19.58 ± 0.04	26.88 ± 0.04	39.58 ± 0.17	<0.001
25(OH)D_2_	0.3 ± 0.68	0.26 ± 0.01	0.35 ± 0.01	0.31 ± 0.01	0.27 ± 0.02	<0.001
25(OH)D_3_	24.16 ± 11.38	11.54 ± 0.05	19.24 ± 0.04	26.57 ± 0.04	39.32 ± 0.17	<0.001

† 25(OH)D concentrations were calculated as the sum of serum 25(OH)D_2_ and 25(OH)D_3_ values. Data are presented as means ± standard deviations or as the number of subjects (percentage). * Categorical variables were analyzed using the Chi-square test, while continuous variables were analyzed using ANOVA to calculate *p*-values. ^abcd^ Different superscript letters denote significant differences between groups (Bonferroni-adjusted *p* < 0.05).

**Table 2:** Multiple regression analysis of serum † 25(OH)D and serum uric acid levels.

Model	Adjusted Variables	Beta Coefficient	*p*-Value	R-Squared
Model 1	Age; Sex	0.0017	0.132	0.28
Model 2	Model 1 + BMI	0.0050	<0.001	0.34
Model 3	Model 2 + Alcohol Use; Cr; Chronic Disease (HTN, DM); Nutritional Intake (Carbohydrate, Fat, Protein); Lipid Levels (HDL-c, TG, LDL-c)	0.0042	<0.001	0.39
Model 4	Model 3 + Dietary Antioxidants (Vitamin C, E, A [RAE]); Aerobic Physical Activity	0.0044	<0.001	0.39

† Serum 25(OH)D concentrations were calculated as the sum of 25(OH)D_2_ and 25(OH)D_3_. Dietary intake of macronutrients (carbohydrate, fat, and protein) and antioxidant vitamins (vitamin C, E, and A [RAE]) was assessed using 24 h dietary recall data. Aerobic physical activity was defined according to WHO guidelines. Sampling weights were applied to ensure population-level representativeness. Covariates were sequentially added across models as indicated.

**Table 3:** Non-linear regression results for serum † 25(OH)D quartiles.

Quartile	Range (ng/mL)	Coefficient	Standard Error	*p*-Value
Q1 (1st quartile)	<15.96	0.292	0.083	<0.001
Q2 (2nd quartile)	15.96–23.2	0.368	0.096	<0.001
Q3 (3rd quartile)	23.2–30.92	0.769	0.217	<0.001
Q4 (4th quartile)	>30.92	0.184	0.251	0.465

† 25(OH)D concentrations were calculated as the sum of serum 25(OH)D_2_ and 25(OH)D_3_ values. All analyses were conducted using sampling weights to ensure representativeness of the study population. Adjusted variables included age, sex, BMI, alcohol use (monthly), creatinine (Cr), chronic disease (hypertension, diabetes), nutritional intake (carbohydrate, fat, protein), and lipid levels (HDL-c, TG, LDL-c).

**Table 4:** Non-linear regression results for serum 25(OH)D_3_ quartiles.

Quartile	Range (ng/mL)	Coefficient	Standard Error	*p*-Value
Q1 (1st quartile)	<15.61	0.265	0.081	0.001
Q2 (2nd quartile)	15.61–22.88	0.351	0.095	<0.001
Q3 (3rd quartile)	22.88–30.61	0.708	0.214	<0.001
Q4 (4th quartile)	>30.61	0.188	0.252	0.456

All analyses were conducted using sampling weights to ensure representativeness of the study population. Adjusted variables included age, sex, BMI, alcohol use (monthly), creatinine (Cr), chronic disease (hypertension, diabetes), nutritional intake (carbohydrate, fat, protein), and lipid levels (HDL-c, TG, LDL-c).

**Table 5:** Non-linear regression results for serum † 25(OH)D quartiles (Model 4).

Quartile	Range (ng/mL)	Coefficient	Standard Error	*p*-Value
Q1 (1st quartile)	<15.96	0.307	0.085	<0.001
Q2 (2nd quartile)	15.96–23.2	0.369	0.099	<0.001
Q3 (3rd quartile)	23.2–30.92	0.837	0.223	<0.001
Q4 (4th quartile)	>30.92	0.274	0.259	0.291

† Serum 25(OH)D concentrations were calculated as the sum of 25(OH)D_2_ and 25(OH)D_3_. All analyses were conducted using sampling weights. Model 4 was adjusted for age, sex, body mass index (BMI), alcohol use, creatinine, chronic diseases (hypertension, diabetes), nutritional intake (carbohydrate, fat, protein), lipid levels (HDL-C, triglycerides, LDL-C), dietary antioxidants (vitamins C, E, and A [RAE]), and aerobic physical activity based on WHO guidelines.

**Table 6:** Subgroup analysis: non-linear regression in older women (≥ 60 years): † 25(OH)D.

Quartile	Range (ng/mL)	Coefficient	Standard Error	*p*-Value
Q1 (1st quartile)	<15.96	0.567	0.210	0.007
Q2 (2nd quartile)	15.96–23.2	0.523	0.157	<0.001
Q3 (3rd quartile)	23.2–30.92	1.281	0.485	0.008
Q4 (4th quartile)	>30.92	0.135	0.262	0.606

† 25(OH)D concentrations were calculated as the sum of serum 25(OH)D_2_ and 25(OH)D_3_ values. All analyses were conducted using sampling weights to ensure representativeness of the study population. Adjusted variables included body mass index (BMI), creatinine (Cr), chronic disease status (hypertension, diabetes), and lipid levels (HDL-c, TG, LDL-c).

## Data Availability

The data presented in this study are available from the Korea National Health and Nutrition Examination Survey (KNHANES) database at https://knhanes.kdca.go.kr (accessed on 20 July 2025). Access to the data requires approval from the Korea Disease Control and Prevention Agency (KDCA).

## Supplementary Materials

The following supporting information can be downloaded at https://www.mdpi.com/article/10.3390/nu17152398/s1.

## Abbreviations

The following abbreviations are used in this manuscript:

BMI	Body Mass Index
WC	Waist Circumference
SBP	Systolic Blood Pressure
DBP	Diastolic Blood Pressure
HbA1c	Hemoglobin A1c
T. Chol	Total Cholesterol
HDL-c	High-Density Lipoprotein Cholesterol
LDL-c	Low-Density Lipoprotein Cholesterol
Hs-CRP	High-Sensitivity C-Reactive Protein
DM	Diabetes Mellitus
HTN	Hypertension
TG	Triglycerides
Cr	Serum Creatinine
UA	Uric Acid
VDR	Vitamin D Receptor
PTH	Parathyroid Hormone
URAT1	Urate Transporter 1
ABCG2	ATP-Binding Cassette Subfamily G Member 2
NPT1/4	Sodium-Phosphate Cotransporter Type 1/4
RCS	Restricted Cubic Spline
