# Supplementary material for: Non-Linear Associations Between Serum Vitamin D and Uric Acid in Korean Adults: 2022–2023 KNHANES Data
Source: Nutrients. 2025 Jul 22;17(15):2398. doi: 10.3390/nu17152398 (PMC12348428; doi:10.3390/nu17152398)
Supplement: Supplementary file 1 [file nutrients-17-02398-s001.zip › nutrients-3717007 - supplementary-update 1.pdf]

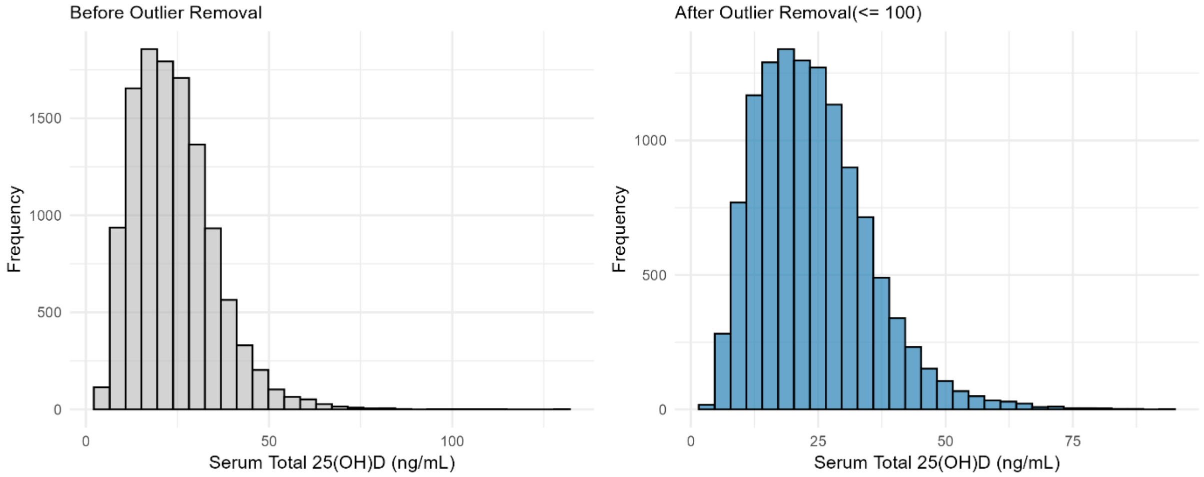

Figure S1. Distribution of serum 25-hydroxyvitamin D [25(OH)D] concentrations before and after outlier removal. Histograms showing the distribution of serum 25-hydroxyvitamin D [25(OH)D] concentrations before and after outlier removal. The left panel depicts the distribution in the full study population, while the right panel shows the distribution after excluding extreme values above 100 ng/mL, based on clinically accepted thresholds for vitamin D toxicity. The overall distribution pattern remained consistent after outlier removal.

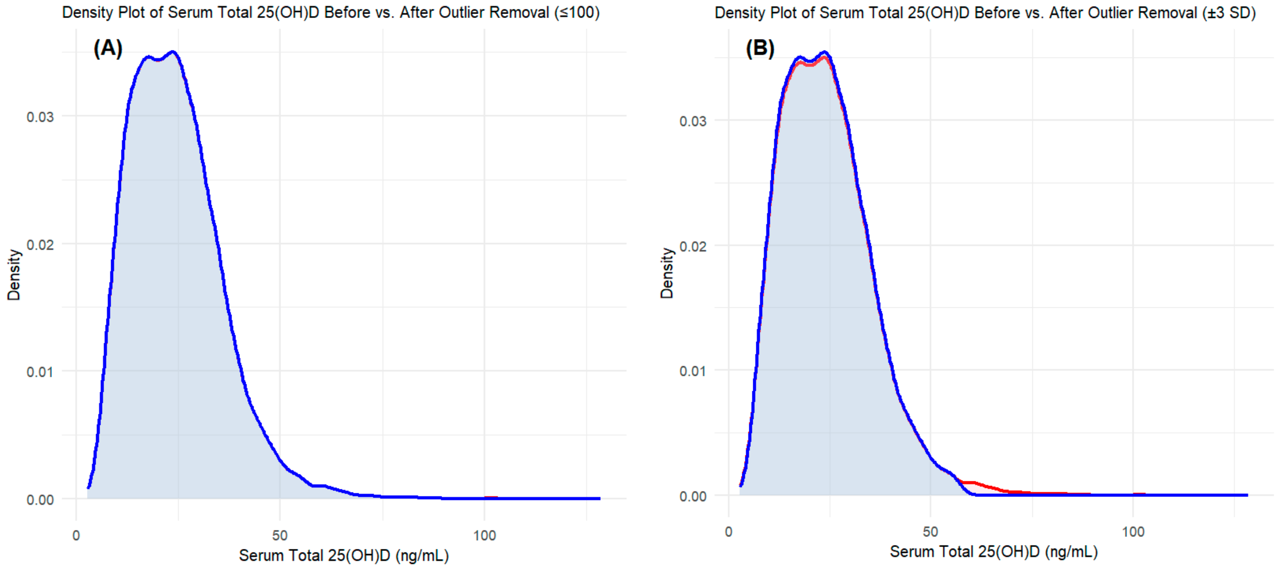

Figure S2. Kernel density plots of serum total 25-hydroxyvitamin D [25(OH)D] concentrations before and after outlier removal using two different criteria.

(A) Density plot comparing the original distribution (red) with the distribution after excluding extreme high values >100 ng/mL based on a clinically accepted threshold.

(B) Density plot comparing the original distribution (red) with the distribution after excluding values beyond ±3 standard deviations (SD) from the mean, based on a statistical definition of outliers.

Table S1. Skewness of serum vitamin D levels before and after log transformation.

Variable	Skewness_Original	Skewness_LogTransformed
†25(OH)D	0.933	-0.335
25(OH)D3	0.946	-0.326

Skewness values were calculated for serum total 25-hydroxyvitamin D [25(OH)D], 25-hydroxyvitamin D3 [25(OH)D_3] to assess distribution symmetry. Log transformation effectively reduced skewness for all variables, bringing their distributions closer to normality.

† 25(OH)D concentrations were calculated as the sum of serum 25(OH)D_2 and 25(OH)D_3 values.

Table S2. Model Comparison (AIC/BIC)

Model	Adjusted variables	AIC	BIC
Model1	Age; Sex	36,864.81	36,901.28
Model2	Age; Sex; BMI	35,332.82	35,376.47
Model3	Age; Sex; BMI; Alcohol Use; Cr; Chronic Disease (HTN, DM); Nutritional Intake (Carbohydrate, Fat, Protein); Lipid Levels (HDL-c, TG, LDL-c)	32,808.31	32,923.93
Model4	Model 3 + Dietary Antioxidants (Vitamin C, E, A [RAE]); Aerobic Physical Activity	30,603.05	30,746.27

Model comparison using Akaike Information Criterion (AIC) and Bayesian Information Criterion (BIC) was performed as follows: Model 1: adjusted for age and sex; Model 2: additionally adjusted for body mass index (BMI); Model 3: additionally adjusted for alcohol consumption, creatinine, chronic diseases (hypertension, diabetes), nutritional intake (carbohydrate, fat, protein), and lipid levels (HDL-C, triglycerides, LDL-C); Model 4: additionally adjusted for dietary antioxidants (vitamins C, E, and A [RAE]) and aerobic physical activity based on WHO guidelines.

Table S3. Comparison of Model Fit Indices (AIC/BIC) by Number and Placement of Knots

Model	Knots	AIC	BIC
3 Knots	15.96, 23.2,30.92	38,050.05	38,108.39
4 Knots	15, 20, 25, 30	38,052.01	38,117.64
5 Knots	15, 20, 25, 30, 35	38,053.99	38,126.92

Comparison of restricted cubic spline regression models with 3, 4, and 5 knots for serum vitamin D levels. Knots were placed at prespecified percentiles, and model fit was evaluated using the Akaike Information Criterion (AIC) and Bayesian Information Criterion (BIC). The model with 3 knots (placed at 15.96, 23.2, and 30.92 ng/mL) showed the best fit based on the lowest AIC and BIC values.

Table S4. Subgroup Analysis of Serum 25(OH)D and Uric Acid by Sex and Age Group

Group	Beta Coefficient	Standard Error	*P*-value	*R*-squared
Male , Age < 60 (n= 2,719)	0.0025	0.0026	0.333	0.148
Female, Age < 60 (n= 3,565)	0.0002	0.0015	0.918	0.133
Male , Age ⩾ 60 (n= 2,014)	0.0020	0.0027	0.455	0.188
Female, Age ≥ 60 (n= 2,561)	0.0042	0.0017	0.016	0.197

Multivariable linear regression analysis of the association between serum vitamin D levels and uric acid concentrations, stratified by sex and age. The regression coefficient (β), standard error (SE), p-value, and coefficient of determination (R^2) are presented for each subgroup. All models were adjusted for body mass index (BMI), serum creatinine, diabetes, hypertension, triglycerides, high-density lipoprotein (HDL), and low-density lipoprotein (LDL) cholesterol.

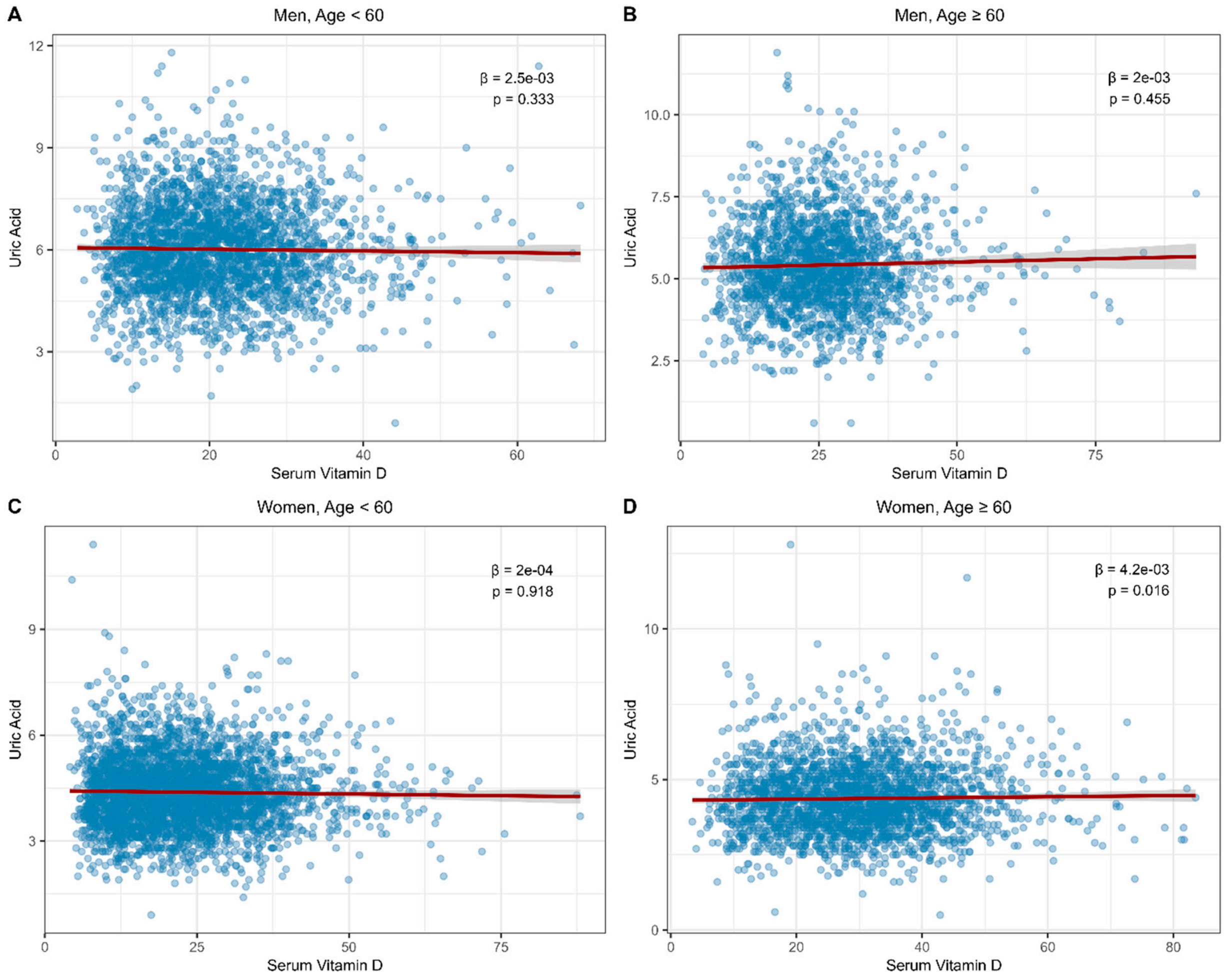

Figure S3. Association between serum vitamin D levels and uric acid concentrations stratified by sex and age groups. Scatter plots with linear regression lines and 95% confidence intervals are presented for each subgroup: (A) Men aged < 60 years, (B) Men aged ≥ 60 years, (C) Women aged < 60 years, and (D) Women aged ≥ 60 years. The regression coefficient (β) and corresponding p-values are indicated in each plot.

Table S5. Subgroup Analysis of Serum 25(OH)D and Uric Acid by Sex and obesity status.

Group	Beta Coefficient	Standard Error	*P*-value	*R*-squared
Male, BMI <25 (n= 2,700)	0.0002	0.002	0.91	0.10
Female, BMI <25 (n= 4,143)	0.001	0.001	0.41	0.08
Male, BMI ≥25 (n= 1,969)	-0.003	0.003	0.32	0.18
Female, BMI ≥25 (n= 1,856)	-0.002	0.002	0.38	0.16

Multivariable linear regression analysis of the association between serum vitamin D levels and uric acid concentrations stratified by sex and obesity status. The regression coefficient (β), standard error (SE), p-value, and coefficient of determination (R^2) are presented for each subgroup. All models were adjusted for body mass index (BMI), serum creatinine, diabetes status, hypertension status, triglycerides, high-density lipoprotein (HDL), and low-density lipoprotein (LDL) cholesterol.

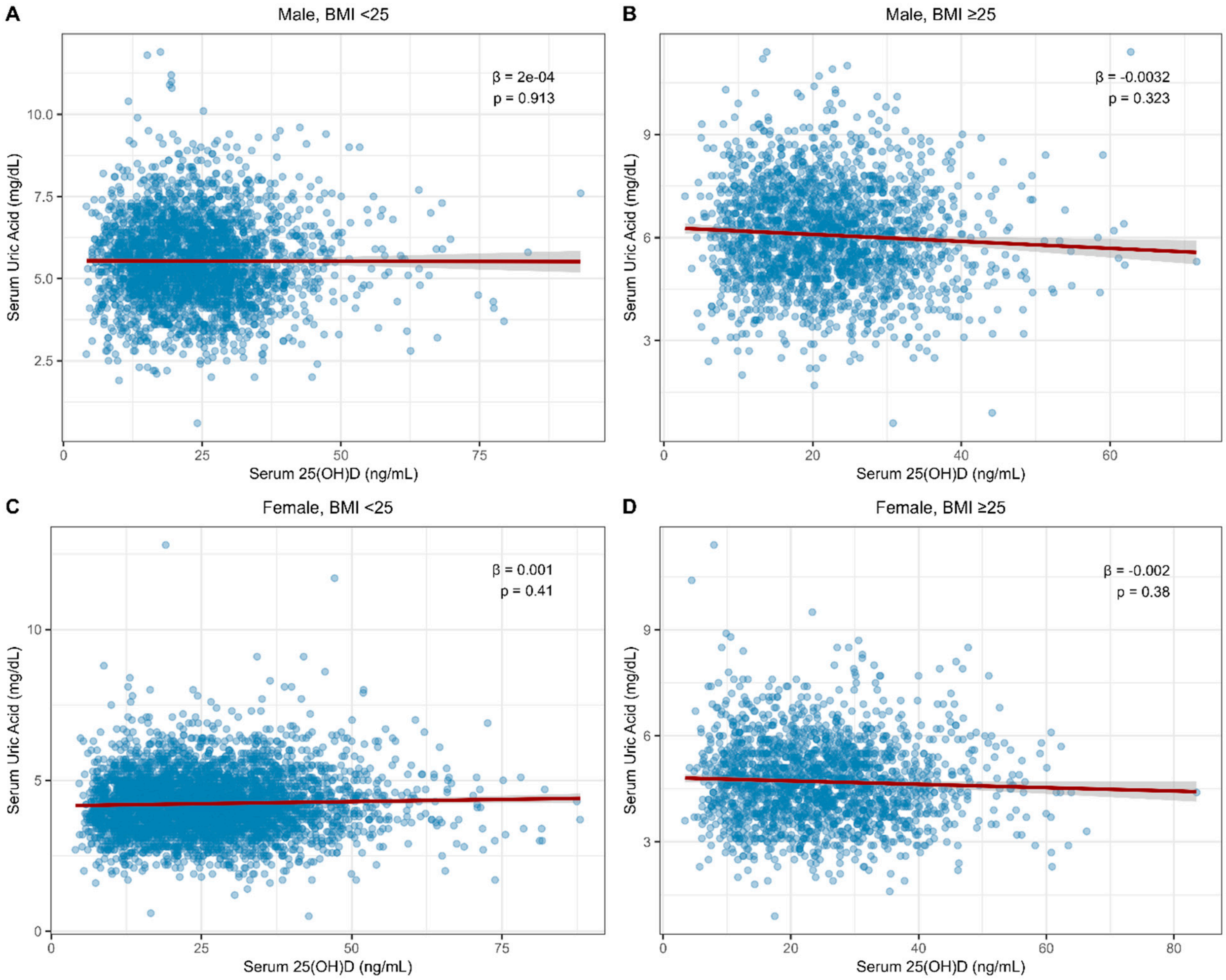

Figure S4. Association between serum vitamin D levels and uric acid concentrations stratified by sex and obesity status. Scatter plots with linear regression lines and 95% confidence intervals are presented for each subgroup: (A) Men with BMI < 25 kg/m², (B) Men with BMI ≥ 25 kg/m², (C) Women with BMI < 25 kg/m², (D) Women with BMI ≥ 25 kg/m². The regression coefficient (β) and corresponding p-values are indicated in each plot.

Table S6. Nonlinear regression results by sex and age subgroup.

Group	Range (ng/ml)	Beta	SE	*P*-value
Male , Age < 60	<15.96	0.3018	0.1548	0.051
Male , Age < 60	15.96–23.2	-0.0463	0.1941	0.811
Male , Age < 60	23.2–30.92	0.7168	0.4125	0.082
Male , Age < 60	>30.92	0.6270	0.3897	0.108
Male , Age ≥ 60	<15.96	0.6069	0.2065	0.003
Male , Age ≥ 60	15.96–23.2	0.2283	0.2252	0.311
Male , Age ≥ 60	23.2–30.92	1.1135	0.5215	0.033
Male , Age ≥ 60	>30.92	0.2238	0.5630	0.691
Female, Age < 60	<15.96	0.0126	0.0904	0.89
Female, Age < 60	15.96–23.2	0.1377	0.1301	0.29
Female, Age < 60	23.2–30.92	-0.1211	0.2591	0.64
Female, Age < 60	>30.92	-0.4516	0.3556	0.204
Female, Age ≥ 60	<15.96	0.5669	0.2099	0.007
Female, Age ≥ 60	15.96–23.2	0.5232	0.1567	<0.001
Female, Age ≥ 60	23.2–30.92	1.2814	0.4854	0.008
Female, Age ≥ 60	>30.92	0.1354	0.2621	0.606

Multivariable spline regression analysis showing the association between serum 25(OH)D_3 concentration ranges and uric acid levels, stratified by sex and age group. Regression coefficients (β), standard errors (SE), and p-values are presented for each serum 25(OH)D_3 concentration range. All models were adjusted for body mass index (BMI), serum creatinine, diabetes status, hypertension status, triglycerides, high-density lipoprotein (HDL) cholesterol, and low-density lipoprotein (LDL) cholesterol.

Table S7. Subgroup analysis : Nonlinear Regression in Older Women (≥ 60 years): 25(OH)D_3

Quartile	Range (ng/ml)	Coefficient	Standard Error	*P*-value
Q1 (1st quartile)	<15.61	0.486	0.202	0.016
Q2 (2nd quartile)	15.61–22.88	0.477	0.153	0.002
Q3 (3rd quartile)	22.88–30.61	1.104	0.469	0.019
Q4 (4th quartile)	>30.61	0.125	0.260	0.632

All analyses were conducted using sampling weights to ensure representativeness of the study population. Adjusted variables included body mass index (BMI), creatinine (Cr), chronic disease status (hypertension, diabetes), and lipid levels (HDL-c, TG, LDL-c).

Table S8. Nonlinear regression results by sex and BMI subgroup.

Group	Range (ng/ml)	Beta	SE	*P*-value
Male, BMI <25	<15.96	0.2330	0.1475	0.1140
Male, BMI <25	15.96–23.2	0.0695	0.1995	0.7280
Male, BMI <25	23.2–30.92	0.7028	0.4075	0.0847
Male, BMI <25	>30.92	0.2794	0.5378	0.6030
Male, BMI ≥25	<15.96	0.2469	0.2049	0.2280
Male, BMI ≥25	15.96–23.2	-0.2821	0.2464	0.2520
Male, BMI ≥25	23.2–30.92	0.3487	0.5468	0.5240
Male, BMI ≥25	>30.92	0.3385	0.5726	0.5540
Female, BMI <25	<15.96	0.1920	0.1007	0.0567
Female, BMI <25	15.96–23.2	0.2275	0.1061	0.0320
Female, BMI <25	23.2–30.92	0.5275	0.2530	0.0371
Female, BMI <25	>30.92	-0.1127	0.2374	0.6350
Female, BMI ≥25	<15.96	-0.1740	0.1714	0.3100
Female, BMI ≥25	15.96–23.2	-0.0590	0.2002	0.7680
Female, BMI ≥25	23.2–30.92	-0.5830	0.4492	0.1950
Female, BMI ≥25	>30.92	-0.4700	0.5207	0.3670

Multivariable spline regression analysis showing the association between serum 25(OH)D_3 concentration ranges and uric acid levels, stratified by sex and BMI Status. Regression coefficients (β), standard errors (SE), and p-values are presented for each serum 25(OH)D_3 concentration range. All models were adjusted for body mass index (BMI), serum creatinine, diabetes status, hypertension status, triglycerides, high-density lipoprotein (HDL) cholesterol, and low-density lipoprotein (LDL) cholesterol.
